# COVID-19 in liver transplant recipients: incidence, hospitalization and outcome in an Italian prospective double-centre study

**DOI:** 10.1038/s41598-022-08947-x

**Published:** 2022-03-22

**Authors:** Maria Guarino, Valentina Cossiga, Ilaria Loperto, Ilaria Esposito, Rosanna Ortolani, Andrea Fiorentino, Giuseppina Pontillo, Lucia De Coppi, Valentina Cozza, Alfonso Galeota Lanza, Giovanni Giuseppe Di Costanzo, Francesco Paolo Picciotto, Filomena Morisco

**Affiliations:** 1 Department of Clinical Medicine and Surgery, Gastroenterology and Hepatology Unit, University of Naples “Federico II”, Via Sergio Pansini, 5, 80131 Naples, NA Italy; 2 UOC Epidemiologia e Prevenzione e Registro Tumori, ASL Napoli 1 Centro, Naples, Italy; 3 Hepatology Unit, AORN A. Cardarelli, Naples, Italy

**Keywords:** Viral infection, Hepatology, Liver diseases

## Abstract

Liver transplant (LT) recipients are vulnerable to SARS-CoV-2-infection (COVID-19), due to immunosuppression and comorbidities. This study aimed to evaluate the impact of COVID-19 on LT recipients compared to general population in the Campania region. In this prospective double-centre study, we enrolled all consecutive adult LT recipients with confirmed SARS-CoV-2-infection. Data were collected at diagnosis of COVID-19 and during follow-up and compared with the regional population. Thirty LT recipients (3.28%) developed SARS-CoV-2-infection (76.66% male, median age 62.61 years). Sixteen (53.33%) were symptomatic. Common symptoms were fever, cough, fatigue, and anosmia. Twenty-five (83.33%) were outpatients, 5 (16.66%) required hospitalization (6.66% admitted to Intensive Care Unit, 6.62% developed Acute Respiratory Distress Syndrome and 6.66% died). Immunosuppressors were in 3 (10%) patients. Incidence rate of COVID-19 was similar between LT patients and general population (3.28% vs 4.37%, p = 0.142) with higher rate of symptoms in LT patients (53.33% vs 15.87%, p < 0.000). At univariate analysis, hospitalization and case fatality rates were higher in LT patients compared to general population (16.66% vs 4.54%, p = 0.001; and 6.66% vs 1.76%, p = 0.041, respectively). At multivariable logistic regression analysis, LT patients with COVID-19 were more frequently symptomatic (OR 5.447 [95% CI 2.437–12.177], p < 0.000), whereas hospitalization and death for COVID-19 were not significatively associated with LT condition (p = 0.724 and p = 0.462, respectively) and were comparable with general population. LT is not a risk factor for acquiring COVID-19. Nonetheless, LT patients are more frequently symptomatic, although comparable to the general population for hospitalization rate and mortality.

## Introduction

The SARS-CoV-2 infection (COVID-19) pandemic rapidly spread in the first months of 2020, becoming a public health matter with several unforeseen challenges to health care systems worldwide and several unmet issues. To date, with 3.258.770 cumulative cases confirmed on January 16th, 2021, Italy is among the most affected countries by the pandemic^1^. Currently, Campania region in Southern Italy is one of the most overwhelmed regions with a cumulative incidence of 5393.79 cases per 100,000 inhabitants^2^.

At present, firm knowledge on disease evolution and optimal management of COVID-19 infection, in the setting of solid organ transplantation are lacking. Transplant recipients are considered as “clinically extremely vulnerable” sub-population for COVID-19 because of lifelong immunosuppressive therapy and their higher rate of underlying comorbidities^3^.

The available data on COVID-19 infection in liver transplant (LT) recipients are not conclusive and are limited to several case series^4–7^, 4 national cohorts from Italy, France, UK and Spain^8–11^ and 3 international studies^12–14^ characterized by marked heterogeneity. However, the lack of non-transplanted patients with COVID-19 as a control-group makes it challenging to assume the increased risk of severe outcomes in liver-transplanted patients.

We collected data from our population of more than 900 LT patients who regularly attended the clinical follow-up program despite the pandemic environment. The current study aims at describing the incidence of SARS-CoV-2 infection, its clinical features at presentation and during disease course, early outcomes, and mortality in a cohort of liver transplanted patients compared to events occurring in the general population (non-transplanted subjects) in the geographical area of Campania Region in Southern Italy.

## Methods

### Study design and target population

This is a prospective double-centre study launched after the outbreak of COVID-19 in Italy on 9th of March 2020. The study was conducted in two regional referral hospital for LT patients (Cardarelli Hospital and Federico II Hospital) in accordance with the Declaration of Helsinki. The protocol was approved by the ethical board of the promoting centre (Federico II Hospital). All data generated or analysed during this study are included in this published article (and its Supplementary Information Files).

All consecutive adult LT patients with concomitant confirmed SARS-CoV-2 infection (with any symptom profile or level of disease severity) were prospectively enrolled in this study at the time of diagnosis of COVID-19 and were followed-up until death or as of as January 16th, 2021. Patients were excluded if they presented any of the following conditions: any recipient of more than 1 simultaneous organ transplant, SARS-CoV-2 infection not confirmed by a nasopharyngeal swab reverse-transcriptase–polymerase-chain-reaction (RT-PCR) test, hospitalisation status or mortality outcome unknown or unreported, or patient aged 18 years or younger at the time of COVID-19 diagnosis.

This collected data regarding COVID-19 infection in our transplanted population was compared with the Campania regional population extracted by National Health System COVID-19 dataset^15^. Our Region accounts for more than 7 million inhabitants and on January 16th, 2021, 308.101 cumulative cases of COVD-19 were registered, with a cumulative incidence of 5393.79 cases per 100,000 inhabitants^1^.

The same inclusion and exclusion criteria have been applied to the population of Campania region with SARS-CoV-2 confirmed infection.

### Diagnostic procedure

COVID-19 infection was confirmed by a RT-PCR assay of nasal and pharyngeal swab specimens, according to the WHO guidelines^16^. In particular, LT patients admitted to the hospital (because of hypoxaemia and/or radiological chest X-ray abnormalities or because of mild symptoms in subjects with significant comorbidities or who were over the age of 60 years) were tested onsite within the first 24 h, whereas outpatients with mild symptoms were tested at home or at ‘drive-through’ testing sites within the next 72 h after contacting a dedicated phone number. For the general population, all symptomatic subjects had been tested at home or at ‘drive-through’ testing sites within the next 72 h from onset of symptoms. Asymptomatic subjects (LT patients and general population) were subjected to nasopharyngeal swab only on the occasion of targeted screening or if they were close contacts of a positive case as required by the ministerial guidelines for the surveillance and control of SARS-CoV-2 infection^16^.

### Collection data

For LT patients, demographic data, comorbidities, information regarding indication to liver transplantation and underlying liver disease, clinical symptoms or signs and laboratory parameters as well as imaging features at the time of COVID-19 diagnosis were collected. Time from liver transplantation was classified as long term (more than 5 years from transplant) and short term (less than 5 years from transplant). Modifications of immunosuppression (reduction or withdrawal; in particular for patients on double immunosuppression, reduction was defined as reduction of at least one immunosuppressive drug and/or discontinuation of one medication, while withdrawal was defined as discontinuation of both drugs) therapy were registered as well as specific drugs (oral hydroxychloroquine or azithromycin, antiviral therapy with lopinavir/ritonavir or remdesivir, steroids or tocilizumab or anti-thrombotic prophylaxis) or ventilation support prescribed for COVID-19.

### Outcomes

The main outcome of the study was the analysis of the burden of COVID-19 infection in a cohort of liver transplanted patients in terms of incidence, type and severity of symptoms at presentation, hospitalisation rate and case fatality rate in comparison to non-transplanted general adult population.

### Statistical analysis

Shapiro–Wilk test has been performed for testing normal distribution. Continuous variables were expressed as median and interquartile range (IQR), and qualitative variables as absolute frequency and percentage. Descriptive statistics of the study variables were performed using Kruskal–Wallis equality-of-populations rank test, Chi-square and Fisher's exact test statistics, as appropriate.

Multivariable logistic regression models were employed to assess the association between LT (dependent variable) and 5 variables (age, gender, symptoms, COVID19 hospitalization and death). In particular, a model has been adjusted for age, gender, COVID19 hospitalization and death to evaluate the impact of SARS-CoV-2 infection on LT (partial model). Afterwards the model was further adjusted for symptoms to evaluate the impact of SARS-CoV-2 disease on LT (Full model).

Statistical analyses were performed using STATA 15 statistical software (StataCorp, College Station, TX, USA).

### Ethics approval

The protocol was approved by the ethical board of the promoting centre (Federico II Hospital) (n. 194/2020).

### Consent to participate

Each enrolled patient provided Informed Consent. No organs were procured from prisoners.

### Consent for publication

In case of acceptance of the manuscript the copyright is transferred to Hepatology International.

## Results

### Characteristics of study population

Among 915 liver transplant (LT) recipients in regular follow-up at the two largest Hepatology Units of the Campania Region in Southern Italy, 30 patients (cumulative incidence: 3.28% [95% CI 3.24–3.32], from 5th January 2020 to 16th January 2021) experienced SARS-CoV-2 infection. Sixteen of them (53.33%) were symptomatic at the time of diagnosis. Clinical characteristics of patients, stratified for the presence of COVID-19 symptoms, are summarized in Table 1. Overall, 23 LT recipients (76.66%) were male and the median age at COVID-19 diagnosis was 62.61 (IQR 52–66) years. Five patients (16.66%) were obese, according to BMI > 30 kg/m^2^. The sex, median age and BMI did not significantly differ between symptomatic and asymptomatic groups. The major indication for LT was hepatocellular carcinoma (HCC) (26.66%), followed by cirrhosis with hepatitis B virus (HBV)/hepatitis delta virus (HDV) infection (16.66% and 23.33%) or hepatitis C virus (HCV) infection (23.33%). Eighteen patients (60%) received a single immunosuppressant agent, with calcineurin inhibitors (CNI) as predominant immunosuppressants (90%). In particular, 17 patients (56.66%) were on tacrolimus (TAC), alone or in combination, 9 (30%) on Cyclosporine A (CsA) alone or in combination, 6 (20%) on mycofenolate mofetil (MMF) alone or in combination and 11 (36.66%) on mTOR inhibitors (everolimus or sirolimus), alone or in combination.

**Table 1:** Baseline characteristics of LT population with COVID-19, overall and according to symptomaticity.

Colonna1	Overall LT COVID-19 cases	COVID-19 asymptomatic cases	COVID-19 symptomatic cases	p value
Patients, n	30	14	16	
Sex, male, n (%)	23 (76.66%)	12 (85.71%)	11 (68.75%)	0.818
Age, years, median (IQR)	62.61 (52–66)	62.64 (55–65)	62.60 (30–68)	0.912*
BMI > 30 kg/mq, n (%)	5 (16.66%)	3 (21.42%)	2 (12.50%)	0.743**
Smoke history, yes, n (%)	4 (13.33%)	4 (28.57%)	0	0.044**
**Time from LT to diagnosis, years, median (IQR)**	12.82 (4–20)	8.62 (1–18)	16.81 (11–27)	0.036*
Short-term LT recipients, n (%)	8 (26.66%)	4 (28.57%)	4 (25%)	0.091**
Long-term LT recipients, n (%)	22 (73.33%)	10 (71.42%)	12 (75%)	0.151**
**Indication to LT**^**§**^**, n (%)**				0.144**
HCV	7 (23.33%)	1 (7.14%)	6 (37.50%)	
HBV	5 (16.66%)	2 (14.28%)	3 (18.75%)	
HBV/HDV	7 (23.33%)	6 (42.85%)	1 (6.25%)	
Alcoholic liver disease	4 (13.33%)	2 (14.28%)	2 (12.50%)	
Biliary Atresia	3 (10%)	1 (7.14%)	2 (12.50%)	
HCC	8 (26.66%)	2 (14.28%)	6 (37.50%)	
Other	4 (13.33%)	2 (14.28%)	2 (12.50%)	
**Baseline immunosuppressive therapy, n (%)**				0.631**
Calcineurin inhibitor	27 (90%)	12 (85.71%)	15 (93.75%)	
Antimetabolite	6 (20%)	1 (7.14%)	5 (31.25%)	
mTor inhibitor	11 (36.66%)	4 (28.57%)	7 (43.75%)	
Single immunosuppressive agent, n (%)	18 (60%)	7 (50%)	11 (68.75%)	0.052**
Two or more immunosuppressive agents, n (%)	12 (40%)	9 (64.28%)	3 (18.75%)	
**Comorbidities, n (%)**				
Cardiovascular disease	10 (33.33%)	6 (42.85%)	4 (25%)	0.605**
Diabetes mellitus	1 (3.33%)	0	1 (6.25%)	0.277**
Active cancer	1 (3.33%)	0	1 (6.25%)	0.277**
Previous Cancer	4 (13.33%)	3 (24.42%)	1 (6.25%)	0.351**
Kidney insufficiency	7 (23.33%)	5 (35.71%)	2 (12.50%)	0.273**
Respiratory disease	4 (13.33%)	1 (7.14%)	3 (18.75%)	0.222**
Two or more comorbidities	15 (50%)	9 (64.28%)	6 (37.50%)	0.464**

Significant values are in bold.

*Kruskal–Wallis equality-of-populations rank test.

**Chi square/Fisher's exact test.

^§^One patient can have more than one cause of liver disease, so the sum of aetiologies did not necessarily sum to 100%.

The median time from LT to COVID-19 diagnosis was 12.82 (IQR 4–20) years and 22 patients (73.33%) had been transplanted more than 5 years before COVID-19 infection. The time from transplant to diagnosis was significantly shorter in asymptomatic (8.62 years) compared to symptomatic group (16.81 years) (p = 0.031). No differences in LT indications, immunosuppressive regimens and comorbidities were found between asymptomatic and symptomatic groups.

In relation to comorbidities, 10 (33.33%) patients had cardiovascular disease, 7 (23.33%) chronic kidney disease and 4 (13.33%) respiratory disease. Concurrent comorbidities were frequent with 15 (50%) patients having two or more comorbidities (Table 1).

### Clinical presentation and outcomes of LT recipients with COVID-19

At the time of COVID-19 diagnosis, the most commonly self-reported symptoms included fever (46.66%), cough (36.66%), muscle pain/asthenia (36.66%), anosmia (36.66%), dysgeusia (33.33%) and dyspnoea (26.66%) (Table 2). Asymptomatic patients were tested for COVID-19 after a high-risk contact, according to surveillance protocols, and had a median recovery time of 13 [IQR 10–21] days, while symptomatic patients of 21 [IQR 17–23] days (p = 0.192).

**Table 2:** Clinical presentation and management of LT patients with symptomatic COVID-19.

Study population	Results
Patients, n	30
**Symptoms at presentation, n (%)**	16 (53.33)
Fever	14 (46.66)
Cough	11 (36.66)
Dyspnoea	8 (26.66)
Fatigue and myalgia	11 (36.66)
GI symptoms	5 (16.66)
Anosmia	11 (36.66)
Dysgeusia	10 (33.33)
**Medications for COVID, n (%)**	
Acetaminophen	14 (46.66)
Steroids	5 (16.66)
Antibiotics	10 (33.33)
Azithromycin	7 (23.33)
> 1 antibiotic	3 (10)
Antivirals	1 (3.33)
Antithrombotic prophylaxis	5 (16.66)
Immunomodulators	0 (0)
Hydroxychloroquine	0 (0)
**Oxygen support, n (%)**	
*Overall*	4 (13.33)
Nasal cannula	2 (6.66)
Invasive ventilation	2 (6.66)
**Modifications of immunosuppression, n (%)**	
*Overall*	3 (10)
Reduction	2 (6.66)
Withdraw	1 (3.33)
**Outcomes, n (%)**	
Hospitalization	5 (16.66)
ICU admission	2 (6.66)
ARDS	2 (6.66)
Death	2 (6.66)

Concerning specific therapies for COVID-19, 14 patients (46.66%) received acetaminophen, 5 (16.66%) steroids, 10 (33.33%) antibiotics (only azithromycin in 7 of them), and only 1 patient (3.33%) received antivirals. Thrombo-prophylaxis, with low molecular weight heparin, was started in 5 patients (16.66%). In our cohort, none of LT recipients needed immunomodulators or hydroxychloroquine. Overall, 4 patients (13.33%) required oxygen support, with nasal cannula in 6.66% and invasive ventilation in 6.66% of LT recipients (see Table 2). Notably, immunosuppressant therapies were modified in 3 patients (all hospitalized) and, particularly, reduction was observed in 2 (6.66%) patients and withdrawal only in 1 patient (3.33%) (see Supplementary Table 1).

Overall, 25 (83.33%) patients received outpatient care for COVID-19, while 5 (16.66%) subjects were hospitalized, with a median duration of hospital stay of 11 [IQR 10–14] days. Among hospitalized patients, 2 (6.66%) LT recipients were admitted to Intensive Care Unit (ICU), 2 (6.66%) developed Acute Respiratory Distress Syndrome (ARDS) and 2 (6.66%) died. General characteristics of hospitalized patients are summarised in Supplementary Table 1. Three out of 5 hospitalized patients had > 60 years and HCC as indication for LT. All of them were long-term transplanted. Moreover, four out of 5 received CNI as single immunosuppressant agent and one of them received combination therapy (CNI + mTOR). Immunosuppressive therapy was modified after COVID-19 diagnosis in 3 patients. Two out of 5 patients required ICU admission, invasive ventilation and died for ARDS after a median time of 7 days. Two out of 30 LT patients died with a crude case fatality rate of 6.66% [95% CI 6.39–6.96].

### Comparison cohort

Among 5.712.143 non-LT subjects in the Campania Region, 233.775 subjects (cumulative incidence: 4.37%, from 5th January 2020 to 16th January 2021) experienced Sars-CoV-2 infection. Half of them (n = 117.321, 50.31%) were males and the median age, at the time of diagnosis, was 44.59 years. In LT recipients the proportion of males was significantly higher than in comparison cohort (76.66% vs 50.31%, p = 0.004), as well as the median age (62.61 vs 44.59 years, p < 0.001). Analysing the clinical outcomes of COVID-19, 196.223 subjects (84.13%) were asymptomatic at the time of diagnosis, with a median recovery time of 20 [IQR 14–27] days. The majority of subjects (95.46%) received outpatient care, while only 7.588 subjects (4.54%) required hospitalization with a median length of 20 [IQR 11–34] days.

**Figure 1:**
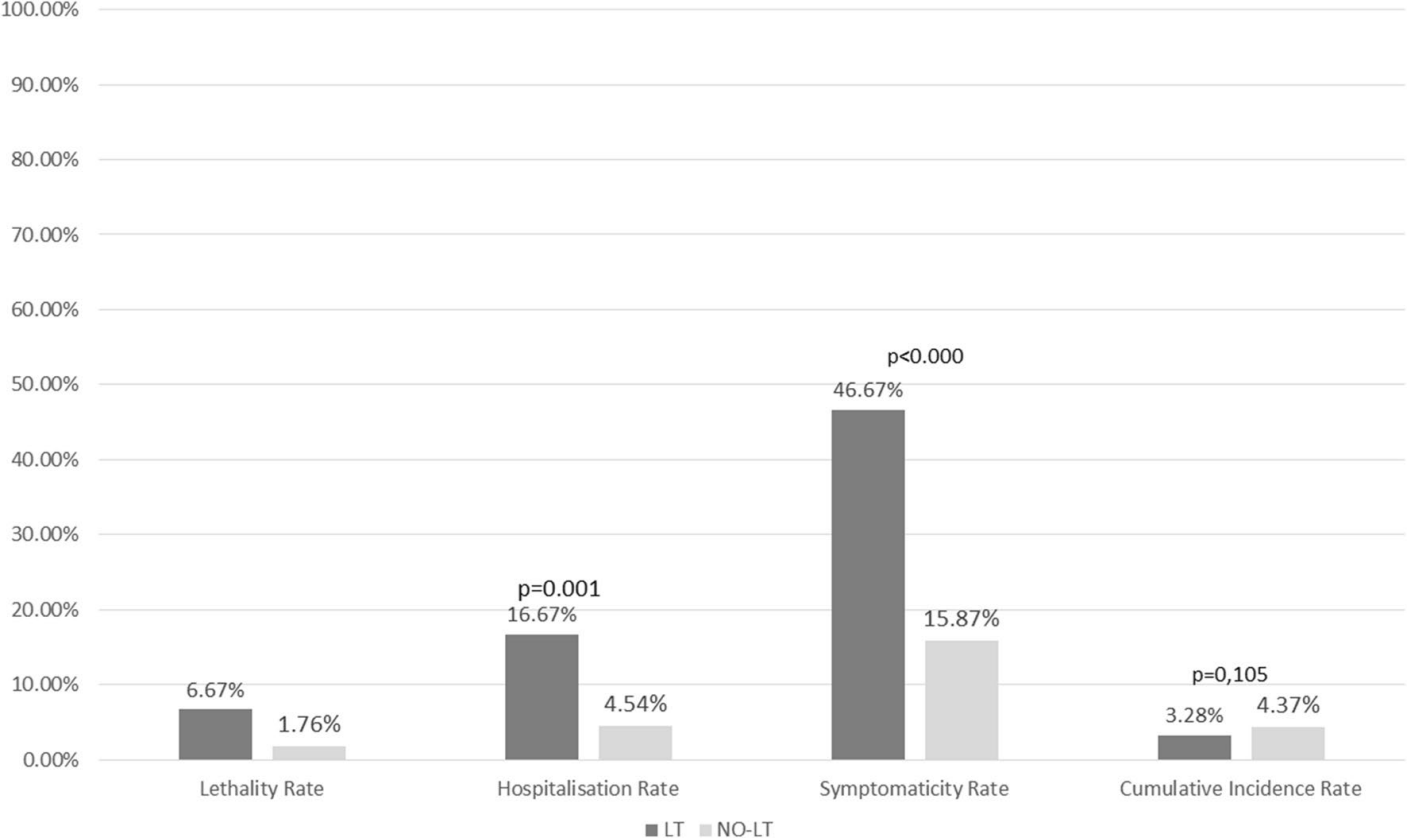
Major outcomes from COVID-19 in LT patients compared to general population within the same geographical area (univariate analysis).

The cumulative incidence rate of COVID-19 and the median recovery time did not differ between LT and non-LT patients (p = 0.112 and p = 0.341, respectively), whereas symptoms were more frequent in the LT population (53.33% vs 15.87%, p < 0.000), see Fig. 1. At univariate analysis, the hospitalization rate was significantly higher in LT recipients than in general population (16.66% vs 4.54%, p = 0.001), but the median length of hospitalization was similar (p = 0.313). Moreover, in the general population of Campania Region, 4.101 (1.76% [95% CI 1.76–1.763) patients died, with a case fatality rate significantly lower than LT subjects (1.76 vs 6.66%, p = 0.041).

Otherwise, at multivariable analysis LT subjects were more frequently male (OR 3.202 [95% CI 1.368–7.494], p = 0.007) and older (OR 1.028 [95% CI 1.008–1.047], p = 0.005) (fully adjusted model). Furthermore, LT patients were more frequently symptomatic (OR 5.447 [95% CI 2.437–12.177], p < 0.000), however hospitalization and death for COVID-19 were not significatively associated with liver transplant neither in the partially nor in the fully adjusted model (p = 0.724 and p = 0.462, respectively) (see Table 3).

**Table 3:** Association between liver transplant, COVID 19—hospitalization, COVID 19—death (X, Y).

	Partially adjusted model				Fully adjusted model			
	OR	95% CI		p	OR	95% CI		p
Age	1.028	1.009	1.048	**0.004**	1.028	1.008	1.047	**0.005**
Sex (M)	3.233	1.381	7.566	**0.007**	3.202	1.368	7.494	**0.007**
Symptomaticity					5.447	2.437	12.177	**0.000**
COVID19 hospitalization	2.445	0.773	7.731	0.128	0.808	0.248	2.633	0.724
COVID19 death	0.689	0.126	3.769	0.668	0.542	0.106	2.767	0.462

Significant values are in bold.

Estimates were derived from multivariable logistic regression models partially and fully adjusted for age, gender, symptomaticity, hospitalization and death.

## Discussion

COVID-19 caused by severe acute respiratory syndrome coronavirus 2 (SARS-CoV-2) infection has been associated with high mortality worldwide. Identifying patients at high risk of infection or those likely to have a severe clinical course is mandatory to guide treatment decisions and infection prevention strategies. In Campania region (Southern Italy) on the 16th of January 2021 the infection rate of SARS-CoV-2 and its case fatality rate have been of 5.39% and 3.15%, respectively^1^. In this scenario, the liver transplant population represents a priori a vulnerable cohort of patients at increased risk of infections and poor outcome due to chronic immunosuppression, high rates of comorbidities and advanced age^17,18^. Whether they are at particularly high risk for critical COVID-19 in real clinical practice still requires further evidence. Herein we report comprehensive data on an Italian regional cohort of LT recipients with confirmed COVID-19 infection, analysing the clinical characteristics, the management and outcome of these patients, and providing a comparison group using patients COVID-19 information of the general population of the same geographical area. Within our study, multivariable analysis of 30 LT patients showed that, despite more symptomatic, they are not at increased risk of COVID-19 infection and of worse outcome in terms of hospitalization and mortality compared to non-transplanted subjects.

The cumulative incidence of SARS-CoV-2 infection in our LT cohort was 3.28%, comparable to the incidence of the general population (4.37%), highlighting that LT patients do not necessarily have a higher risk of infection. This first result was in line with data reported by Bhoori et al.^4^ showing an incidence rate of 3% in long term transplanted patients, and with data by Mocchegiani et al.^19^ showing an incidence rate of COVID-19 infection in LT patients (0.87%) similar to the general population of Marche Region (0.44%). Conversely, our results contrast with Trapani et al.^8^ that reported an incidence rate in LT patents three times higher than that estimated for the Italian population.

In terms of clinical presentation, in our cohort of LT patients, almost 50% showed a symptomatic course, a percentage decisively higher than that observed in the control group (53.33% vs 15.87%). These observations are distinct from the data reported by Colmenero et al.^11^ showing a symptom rate of 93% in LT subjects. No other studies published until now reported this information, since most of them were focused only on symptomatic cohort of LT patients^5,8–10,12–14^. Moreover, the clinical presentation of COVID-19 infection in LT recipients is similar to that observed in non-transplanted population, with fever, fatigue, anosmia and cough being the four more common symptoms. These findings are sustained also by Dumortier et al.^9^ and Becchetti et al.^14^, both showing that fever and cough were the most common symptoms in LT population, while anosmia was present only in 10% of patients.

Based on our results, the management at home of the majority of COVID-19 positive LT patients seems to be possible with good outcomes and limiting the need of hospitalization (in our cohort only 16.66%). In our experience, we decide for hospitalisation on a single case-basis according to a close monitoring of clinical conditions (indication to hospitalisation in case of worsening of respiratory symptoms with hypoxaemia and/or radiological chest X-ray abnormalities or because of mild symptoms in subjects with significant comorbidities or who were over the age of 60 years). For the group of patients managed at home, teleconsultation or phone call surveillance until disease resolution was offered^20^. Our Hepatology Unit recognised the pandemic challenges early and implemented several preventative measures for minimizing in-person visits. Teleconsultations, like a fully implemented telemedicine service, partially restructured for the COVID-19 pandemic with video-consultations were prioritized, as well as, extended helpline hours and smart working from home to increase remote patient monitoring and mobile health care. Concomitantly, these measures reduced the risk due to contact with medical staff^20^.

It has been described that more than 50% of LT patients develop severe forms of COVID-19 disease^21^. Fortunately, our study does not support this observation. Despite well-known risk factors for poor outcome of COVID-19 infection such as chronic immunosuppression, high rates of comorbidities and advanced age, COVID-19 infection in our LT patients showed a clinical course not more severe than that observed in general population as also reported by Becchetti et al.^14^. This result is probably related to several factors: the younger median age in our LT cohort (62.61 vs > 65 years) compared to other studies^11,14^, the higher median time from LT to COVID-19 diagnosis (12.82 vs < 10 years)^11,12,14^ and the lower prevalence of comorbidities (50% vs 79% of LT patients with one comorbidity)^12^ compared to other cohorts.

Conversely, even if the role of comorbidities as relevant risk factors for COVID-19 mortality has been demonstrated in the general population^22^ and despite being highly prevalent among LT patients, in the present study no specific comorbidity, nor a combination of comorbidity resulted independently associated with worse clinical outcome. This agrees with the results reported by Belli et al.^12^.

The management of immunosuppression in LT patients with COVID-19 is a matter of concern in scientific communities with a large debate on the opportunity to decrease or swich drugs. In our cohort, the majority of patients with COVID-19 were kept on their usual immunosuppressive regimen without need of modifications [drugs modifications only for 3 patients, all hospitalised for severe COVID-19 disease]. On the other hand, in 50% of our LT patients who required hospitalisation, immunosuppressive treatment was modified, according to the experience reported in literature showing a need to decrease immunosuppression in severe and critical conditions^4,7,9,14^. Nonetheless it is relevant to underline that therapy for COVID-19 differs across centres and countries and varied overtime with the increasing knowledge acquired. In our experience, the most used drug was acetaminophen, with a lower use for steroids and antibiotics in comparison to other published studies^7,11,14^. Finally, regarding the positive role of tacrolimus, as antivirals, in the treatment of COVID-19 disease as suggested by some reports, since 90% of our patients were treated with CNIs, it was not possible to assess any specific effect of this drug.

The hospitalization rate in this study was 16.66%, a rate extremely lower than the rates reported in other series [range varying from 71 to 86.5%]^7,8,11–14^. The explanation of this discrepancy is presumably due to the period of COVID-19 infection; in fact, all COVID-19 infections in our cohort developed during the second wave of the pandemic (from September 2020 to January 2021) when knowledge and abilities in the management of the infection in immunosuppressed patients improved, reflecting a better awareness of patients and a lower clinician anxiety regarding the uncertain impact of COVID-19 disease course in liver transplant recipient. Indeed, all the published studies until now, analysed cases of COVID-19 infection in LT patients in the first months of 2020 during the first wave of pandemic (most of them from March to May—^7,11,12,14^—and not later than September 2020^8^).

The overall case fatality rate of SARS-CoV-2 infection in our LT cohort was 6.62%, significantly higher than that observed in regional general population (1.76%), but decisively lower than the mortality rates reported by literature ranging from 10 to 27.3%^4,7,8,10–14^ and by a recent review reporting a cumulative mortality rate of 20%^23^. To analyse these results, it is necessary to consider that the case fatality rate in Campania was always lower than in the other Italian regions (1.54% vs 3.99%), probably due to the presence of a younger population. Conversely, the mortality rate in hospitalized patients resulted higher (40%) and in line with reported findings showing a rate ranging from 17 to 29%^7,9,14^.

This study has some strengths. First of all, we provided a large control group of non-transplanted subjects living in the same geographical area according to the epidemiology of the COVID-19 infection; this permits a better characterization of the impact of COVID-19 in patients with LT and strengthens the argument that these patients are not at higher risk of adverse COVID-19 outcomes. Second, our study is a prospective study and not a survey, with prospective acquisition of data collected in 2 centers with high levels of expertise in hepatology and infectious diseases. Third, we can give a picture of the real incidence of COVID-19 infection in LT recipients because, during the study period we contacted or visited in-person all the LT patients followed-up at our centers to be sure they were not infected or have suspicious of infection until the end of the study (16th of January 2021). Finally, this is the first study providing data on LT patients with asymptomatic COVID-19 infection.

Nonetheless some potential limitations are also to be acknowledged. Firstly, the small sample size (30/915 LT patients), even if picturing the epidemiological situation of a well-defined geographical area, can limit the interpretation of the results. Second, some information relating to the comorbidities of COVID-19 positive subjects is not routinely collected in the NHS-ISS dataset. For this reason, the characterization of the two populations appears unbalanced with a lack of clinical information relating to non-LT subjects.

## Conclusions

We can assert that LT patients do not appear to be more prone to COVID-19 infection and, although they more often exhibit symptoms, they are not at increased risk of adverse outcomes in terms of hospitalization or mortality when compared to the general population. The authors believe that, although this population is more fragile, early diagnosis, careful and personalized home management in patients accustomed to the responsible relationship with the healthcare professional have a positive influence on the outcomes.

## Supplementary Information

Supplementary Information.

## Abbreviations

COVID-19: SARS-CoV-2 infection
LT: Liver transplant
RT-PCR: Reverse-transcriptase-polymerase-chain-reaction
CI: Cumulative incidence
IQR: Interquartile range
HCC: Hepatocellular carcinoma
HBV: Hepatitis B virus
HDV: Hepatitis delta virus
CNI: Calcineurin inhibitors
TAC: Tacrolimus
CsA: Cyclosporine A
MMF: Mycofenolate mofetil
ICU: Intensive care unit
ARDS: Acute respiratory distress syndrome
