# Supplementary material for: COVID-19 in liver transplant recipients: incidence, hospitalization and outcome in an Italian prospective double-centre study
Source: Sci Rep. 2022 Mar 22;12:4831. doi: 10.1038/s41598-022-08947-x (PMC8940902; doi:10.1038/s41598-022-08947-x)
Supplement: Supplementary file 1 — Supplementary Information. [file 41598_2022_8947_MOESM1_ESM.docx]

**Supplementary Table 1.** Case summaries of hospitalized LT patients with COVID-19, including demographics, immunosuppression details, treatment and outcomes.

|  | **Sex** | **Age (years)** | **BMI > 30 Kg/m**² | **Indication to LT** | **Time interval from liver transplant**  **(years)** | **Comorbidities** | **Smoke history** | **Clinical presentation** | **Baseline immunosuppression** | **Immunosuppression changes** | **COVID-19 Medications** | **Respiratory support** | **ICU admission** | **Outcomes** |
| --- | --- | --- | --- | --- | --- | --- | --- | --- | --- | --- | --- | --- | --- | --- |
| 1 | M | 49 | YES | HBV/HDV | 10 | Obesity, CDK | yes | Pneumonia, Fever, dyspnoea, cough | CNI | reduction | steroids, > 1 antibiotics, anti-thrombotic | none | no | discharged after 25 days |
| 2 | M | 55 | NO | HBV | 22 | None | no | Fever, fatigue, myalgia, dyspnoea, cough, diarrhoea, anosmia, dysgeusia | CNI | none | steroids, Azithromycin, anti-thrombotic | none | no | discharged after 14 days |
| 3 | M | 75 | NO | HCC | 18 | Active Lung cancer | no | Fever, fatigue, myalgia, dyspnoea, cough | CNI | none | steroids, Azithromycin, anti-thrombotic | nasal cannula | no | discharged after 11 days |
| 4 | M | 79 | NO | HCC | 20 | Cardiovascular disease, COPD | no | Pneumonia, Fever, fatigue, myalgia, dyspnoea, cough, anosmia, dysgeusia | CNI | withdraw | steroids, > 1 antibiotics, anti-thrombotic | invasive mechanical ventilation | yes | **death after 4 days for ARDS** |
| 5 | M | 63 | NO | HCC | 10 | Cardiovascular disease | no | Pneumonia, Fever, fatigue, myalgia, dyspnoea, cough, anosmia, dysgeusia | CNI+ mTOR | reduction of both drugs | steroids, > 1 antibiotics, anti-thrombotic | invasive mechanical ventilation | yes | **death after 10 days for ARDS** |
